# Mechanical Strain-Mediated Tenogenic Differentiation of Mesenchymal Stromal Cells Is Regulated through Epithelial Sodium Channels

**DOI:** 10.1155/2020/5385960

**Published:** 2020-08-18

**Authors:** Hui Yin Nam, Malliga Raman Murali, Raja Elina Ahmad, Belinda Pingguan-Murphy, Hanumantha Rao Balaji Raghavendran, Tunku Kamarul

**Affiliations:** ^1^Tissue Engineering Group, Department of Orthopaedic Surgery (NOCERAL), Faculty of Medicine, University of Malaya, 50603 Kuala Lumpur, Malaysia; ^2^Department of Physiology, Faculty of Medicine, University of Malaya, 50603 Kuala Lumpur, Malaysia; ^3^Department of Biomedical Engineering, Faculty of Engineering, University of Malaya, 50603 Kuala Lumpur, Malaysia

## Abstract

It has been suggested that mechanical strain may elicit cell differentiation in adult somatic cells through activation of epithelial sodium channels (ENaC). However, such phenomenon has not been previously demonstrated in mesenchymal stromal cells (MSCs). The present study was thus conducted to investigate the role of ENaC in human bone marrow-derived MSCs (hMSCs) tenogenic differentiation during uniaxial tensile loading. Passaged-2 hMSCs were seeded onto silicone chambers coated with collagen I and subjected to stretching at 1 Hz frequency and 8% strain for 6, 24, 48, and 72 hours. Analyses at these time points included cell morphology and alignment observation, immunocytochemistry and immunofluorescence staining (collagen I, collagen III, fibronectin, and N-cadherin), and gene expression (ENaC subunits, and tenogenic markers). Unstrained cells at similar time points served as the control group. To demonstrate the involvement of ENaC in the differentiation process, an ENaC blocker (benzamil) was used and the results were compared to the noninhibited hMSCs. ENaC subunits' (*α*, *β*, *γ*, and *δ*) expression was observed in hMSCs, although only *α* subunit was significantly increased during stretching. An increase in tenogenic genes' (*collagen1*, *collagen3*, *decorin*, *tenascin-c*, *scleraxis*, and *tenomodulin*) and proteins' (collagen I, collagen III, fibronectin, and N-cadherin) expression suggests that hMSCs underwent tenogenic differentiation when subjected to uniaxial loading. Inhibition of ENaC function resulted in decreased expression of these markers, thereby suggesting that ENaC plays a vital role in tenogenic differentiation of hMSCs during mechanical loading.

## 1. Introduction

Ion channels have been regarded as an important mediator for a multitude of physiological processes including muscle contraction, synaptic transmission, immune regulation, and many others [1, 2]. It is therefore not surprising that these structures are also involved in specific cellular responses including cell cycle regulation, cytoskeletal reorganization, and apoptosis [3, 4]. Whilst many of the common ion channels have been extensively investigated, the role of the less common ones has been underrated. This has led to the lack of understanding of the mechanism regulating specific cellular function involving these channels such as the signalling process in response to mechanical stimuli. Amongst the less commonly studied ion channels is the epithelial sodium channels (ENaC), which has been reported to have a main role in facilitating movements of fluids across the cells mainly in the lungs, kidneys, and skin [5, 6].

Several studies have reported that ion channels residing in the plasma membrane of chondrocytes and osteoblasts are involved in the transduction of mechanical signals [7–9]. The existence of ENaC in load-bearing cells suggests that ENaC has a mechanoactive role in cellular signalling. Such signalling processes are deemed important for cellular differentiation to occur. Although the differentiation process of adult somatic cells is thought to be mediated by ENaC, its mechanoactive role in multipotent cells, such as those of mesenchyme origin, has not been previously described. Furthermore, whilst it has been shown that ENaC activation occurs via mechanical stretching, the mechanism resulting in the sequelae of events has never been fully understood.

It is suggested that ENaC functions as transmembrane adhesion molecules that is linked directly to the cytoskeletal microtubules as well as the extracellular matrix (ECM) components such as collagen type IV [10]. As proposed by Shakibaei and Mobasheri [11], ECM macromolecules (collagen type II), *β*1-integrins, ENaC, and voltage activated calcium channel (VACC) act as putative mechanosensitive receptors that regulate subcellular signal transduction pathways through the perception of physical contact and stresses from the ECM. It is also suggested that the ability of cells to respond to mechanical stimuli is controlled by a series of mechanosensitive receptors or structures that sense and convert mechanical signals into biochemical signalling events. This eventually leads to the control of cellular functions, which include but not limited to cell proliferation, differentiation, and apoptosis. This process, known as mechanotransduction, is deemed to be mediated by sodium currents and thus can be controlled through sodium channels [12]. ENaC activity can be inhibited by potent pharmacological blockers such as benzamil, which disrupts the mechanical transduction process and signalling pathways that would result in cellular activity [13, 14]. Thus, the use of such inhibitor provides an opportunity to study the functional role of ENaC in transducing mechanical stimuli into cell responses.

Mesenchymal stromal cells (MSCs), being undifferentiated and having multipotent differentiation ability, have a tremendous potential for various biomedical and therapeutic applications in the field of regenerative medicine [15, 16]. The application of *in vitro* differentiation of MSCs into tissue progenitors prior to transplantation circumvents the development of ectopic tissue or tumour formation *in vivo* and, in many studies, demonstrates superior tissue repair outcomes [17]. Current strategies to direct tenogenic differentiation of MSCs generally involve physical manipulations as well as treatment with various biochemical factors; these include mechanical stimulation, the use of scaffolds, administration of growth and differentiation factors, gene transfection, and coculture with specific tissues or cells [18–20]. The use of mechanical loading provides a viable alternative [21–23] to enhance cellular differentiation as it simulates the natural stimuli the cells would be exposed to *in vivo* such as the loading that occurs during load-bearing activities of daily living [24]. Indeed, the application of mechanical stimuli with or without scaffolds or growth factors may be an effective strategy to enhance the expression of tendon-specific markers and induce stability of the tenogenic phenotype. However, the mechanisms regulating tenogenic differentiation of MSC induced by mechanical loading remain elusive. Several studies have suggested that ENaC may play an important role in this. This knowledge is important, as the control of ENaC function may lead to better regulation of the tenogenic differentiation process and, indirectly, of tendon regeneration. To establish this, we conducted a study to investigate the mechanoactive role of ENaC in regulating tenogenic differentiation of MSCs, using benzamil to inhibit ENaC function. We hypothesise that ENaC regulates tenogenic differentiation of MSCs and that the restriction of sodium supply induced by ENaC inhibition during cyclical tensile loading will affect the mechanical strain-induced tenogenic differentiation of MSCs and the resultant ECM production by the cells.

## 2. Materials and Methods

### 2.1. Harvesting Bone Marrow Specimens from Human

Experiments using human bone marrow-derived mesenchymal stromal cells (hMSCs) were conducted following the approval from the Medical Ethics Committee in University Malaya Medical Centre (reference number: 369.19). Two assigned orthopaedic surgeons were tasked with the job of harvesting bone marrow specimens from patients undergoing knee replacement procedures using a large aspirator. This was done after obtaining written informed consents from 10 patients (*N* = 10; mean age = 65.1 ± 3.07 years). Samples were obtained from either the femur or tibia of these patients.

### 2.2. Culture of hMSCs

**Figure 1:**
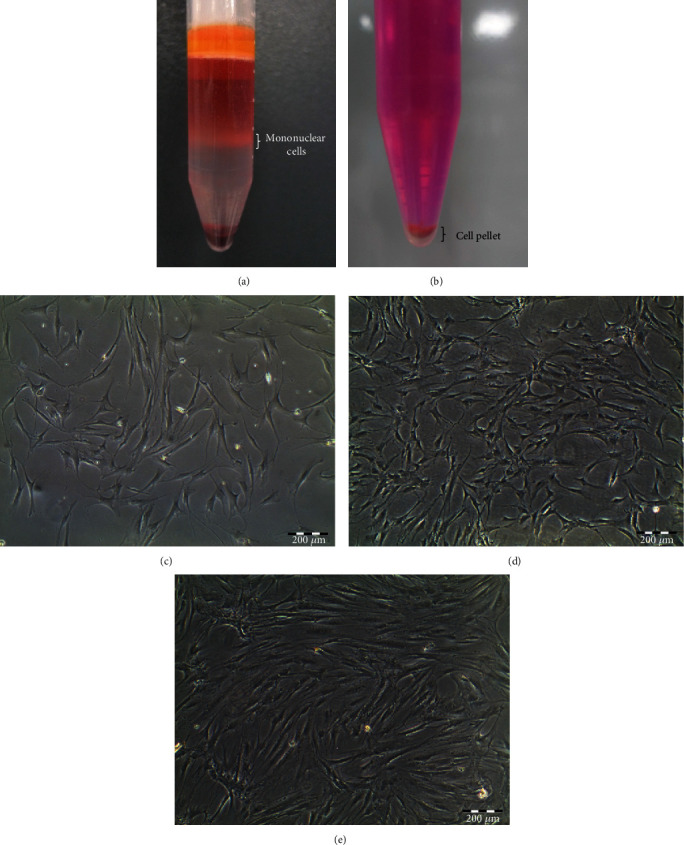
Photomicrographs of human bone marrow-derived MSCs. (a) Mononuclear cells were extracted after density centrifugation. (b) The cell pellet which contains hMSCs was formed and cultured. (c) The primary cultures of the passaged-0 cells contained fibroblastic cells at day 9. (d) Passaged-1 hMSC morphology at day 12. (e) Passaged-2 hMSC morphology at day 14.

An equal volume of pH 7.2 phosphate-buffered saline (PBS) (Invitrogen-Gibco, Grand Island, NY, USA) was added into bone marrow specimens and slowly layered on top of the 3 mL of the density of 1.077 g/mL Ficoll-Paque PREMIUM (Amersham Biosciences, Uppsala, Sweden). Centrifugation at 2,200 rpm for 25 min was then performed. The mononuclear cells (see Figure 1(a)) were extracted and washed with low-glucose Dulbecco's modified Eagle's medium (DMEM) (Invitrogen-Gibco, USA) and underwent centrifugation at 1,600 rpm for 10 min. The supernatant was then discarded, and the cell pellet (see Figure 1(b)) formed at the bottom was resuspended using 1 mL of fetal bovine serum (FBS) (Invitrogen-Gibco, USA). Cell count and viability test were performed. The mixture of mononuclear cells was then cultured in cell culture medium, which consisted of DMEM, 10% FBS, 1% GlutaMAX-1, and 1% penicillin-streptomycin (Invitrogen-Gibco, USA). Cultures were maintained at 37°C in a humidified atmosphere containing 5% CO_2_. Suspended cells were discarded after 5 days of culture, and adherent cells were left to grow on the flask surface. Culture medium was changed every 3 days until the cultures became 75% to 80% confluent. In order to obtain a sufficient number of hMSCs, the cells were serially passaged and expanded up to passage-2 (see Figures 1(c)–1(e)) before being used for experiment use. The hMSCs used in our study were well-characterized by flow cytometric analysis and induction of multilineage differentiation assay, according to the previous protocols used in our laboratory [25, 26].

### 2.3. Benzamil Treatment on hMSCs

A stock solution 10 mM of benzamil (Sigma, USA) was prepared in 100% methanol. To optimize the concentration of benzamil to be used in this study, benzamil at various concentrations (1 *μ*M, 10 *μ*M, 25 *μ*M, 50 *μ*M, and 100 *μ*M) was diluted with culture medium immediately before the treatment on hMSCs was performed. The cell morphology was observed, and images captured after 72 h were being treated with benzamil.

### 2.4. Cell Seeding and Application of Mechanical Stretching

hMSCs from the second passage in culture were harvested and counted, and an overall viability of more than 90% was observed using a trypan blue (Invitrogen-Gibco, USA) exclusion test. A total of 10^5^ hMSCs were plated on each collagen type I-coated (Sigma, USA) silicone chamber (STREX, Japan). After 48 h of culture, the concentration of FBS was reduced to 1% for 24 h in order to align most cells into the G_0_ phase of the cell cycle and changed to growth medium with or without 10 *μ*M benzamil, before assembling into a uniaxial strain device. A commercial instrument (Model ST-140, STREX Co., Ltd., Osaka, Japan) was used to conduct experiments to determine the effects of cyclic uniaxial strained on hMSCs. Uniaxial strain was applied in order to imitate the physiological stretching conditions for tendons and ligaments *in vivo*. Uniaxial cyclic stretching at a frequency of 1 Hz and a magnitude of 8% was applied. This setting was used based on our previous findings which demonstrated an enhanced collagen synthesis or tenogenesis gene expression [23]. Cells in the control group also were cultured on a silicone chamber and maintained in the same incubator but without stretching. The cells were harvested after 6, 24, 48, and 72 h of cyclic loading for downstream experiments, including microscopy of cells, immunostaining (72 h), and gene expression assay.

### 2.5. Collagen Immunohistochemistry

Collagen staining was performed according to the manufacturer's recommendation (Dako, Denmark). The methanol-fixed unstrained and strained cells were applied using hydrogen peroxidase to reduce nonspecific background for 5 min. Primary antibodies, i.e., rabbit anti-collagen type I or rat anti-collagen type III (Merck, USA), were diluted at 1 : 100 and were applied to each specimen and incubated for 30 min. Subsequently, the specimens were incubated with streptavidin-peroxidase secondary antibody (Dako, Denmark) for 30 min. For signal detection, 3,3′-diaminobenzidine tetrahydrochloride chromogen substrate was applied for 5 min and examined under light microscopy (Nikon Eclipse TE2000-S; Nikon Corporation, Japan).

### 2.6. N-Cadherin and Fibronectin Immunofluorescence Staining

hMSCs were fixed with 3.7% paraformaldehyde in PBS, followed by permeabilization with -20°C acetone, and incubated with 1% bovine serum albumin to block nonspecific binding of antibodies. For N-cadherin and fibronectin staining, the specimens were incubated with respective primary antibodies (Abcam, UK) diluted at 1 : 300 for 1 h and with appropriate FITC secondary antibodies (Abcam, UK) diluted at 1 : 600 for 1 h. Nuclei were stained by Hoechst (Molecular Probes, USA) in blue. The fluorescently stained samples were imaged by using a laser scanning confocal microscopy system (Leica TCL SL, Germany).

### 2.7. RNA Isolation and Multiplex Gene Expression Assay

To determine the correlation between the effects of hMSCs by mechanical stimulation and ENaC blocking activity, we used multiplex gene expression assay. Total RNA was extracted from unstrained and strained hMSCs using the RNeasy mini kit (Qiagen, Canada). RNA concentration and purity were assessed using a NanoDrop Spectrophotometer (ND-1000, NanoDrop Technologies, Wilmington, DE), and RNA integrity was assessed with a BioAnalyzer (Model 2100, Agilent Technologies). Only samples with high quality were selected for microsphere-based multiplex branched DNA downstream analysis. The mRNA expression of tenogenic lineages and ENaC subunits (see Table 1) was quantified by the QuantiGene 2.0 Plex assay (2.0 plex set 12082, Panomics/Affymetrix Inc., Fremont, CA, USA). The housekeeping gene was *PGK1* (phosphoglycerate kinase 1), which has been observed in our previous pilot study [26].

### 2.8. Statistical Analysis

The assays were carried out with a minimum number of technical triplicates (*n* = 3) per experimental run, using six independent samples from different donors (*N* = 6) for each group of the experiment. Data were presented as the mean ± standard deviation (SD). Statistical significance was analysed using one-way analysis of variance (ANOVA). A probability value (*p* value) of less than 0.5 was deemed to be statistically significant.

## 3. Results

### 3.1. Baseline Expression of ENaC Subunits in hMSCs

**Figure 2:**
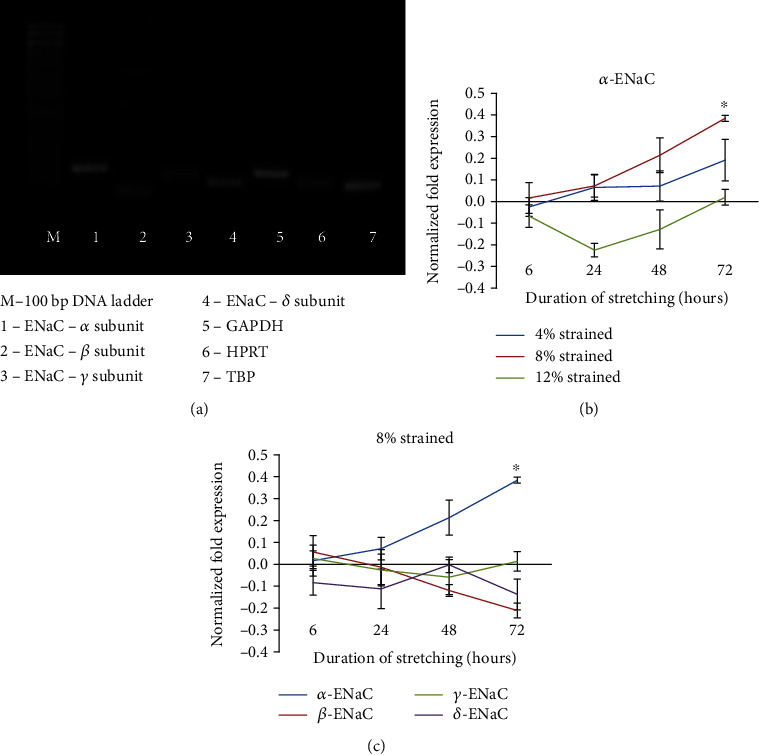
The expression of the *α*-, *β*-, *γ*-, and *δ*-ENaC mRNA in unstrained and strained hMSCs. (a) Analysis of RT-PCR products from hMSCs indicating the presence of the ENaC subunits. (b) mRNA expression of hMSCs shows *α*-ENaC expressed highest in 8% strained compared with 4% and 12% strained. (c) Expression of ENaC subunits at 8% strained at 1 Hz. Fold changes of expression were counted by normalizing to the relative expression amount of corresponding control groups (unstrained groups). Statistical significance (*p* < 0.05) was represented by ∗ which compared to unstrained. Error bars represent the SD of the mean of six biological replicates.

Semiquantitative PCR was performed to identify the presence of *α*, *β*, *γ*, and *δ* subunits of ENaC, and it was found that all four subunits are expressed in hMSCs (see Figure 2(a)). On using different strain magnitudes, it was observed that 8% strained cells expressed higher *α* subunit expressions as compared to 4% and 12% strain (see Figure 2(b)). Upon subjecting the cells to stretching at 1 Hz + 8%, the expression of *α* subunit increased significantly over time. However, there were no changes in the genes expression of the *β*, *γ*, and *δ* subunits (see Figure 2(c)).

### 3.2. Morphology of ENaC-Inhibited hMSCs after Mechanical Stimulation

**Figure 3:**
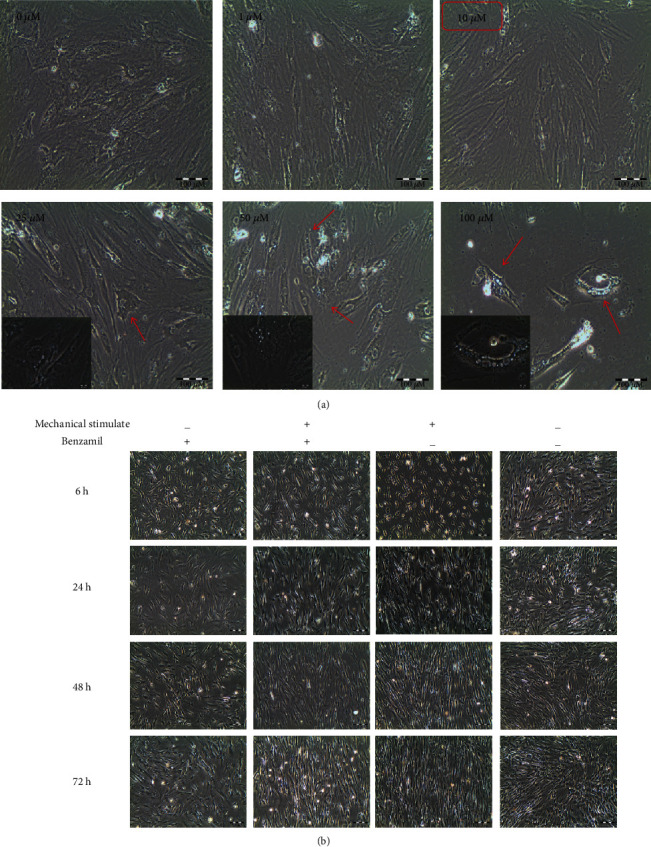
Morphology of hMSCs after treatment with benzamil. (a) Morphological changes of hMSC cell culture after 72-hour incubation with benzamil. Increasing concentration of benzamil resulted in the appearance of small vesicles (probably apoptotic bodies, see arrow). (b) Morphology of the unstrained and strained cells at 1 Hz, 8%, at different durations of exposure to mechanical stretching, with or without administration of 10 *μ*M benzamil, respectively.

Unstrained hMSCs were treated with different concentrations of benzamil (1, 10, 25, 50, and 100 *μ*M) to identify the optimal concentration of benzamil that can be used in the study without causing morphological changes or cell detachment (see Figure 3(a)). Cells treated at the concentration of 1 *μ*M and 10 *μ*M showed normal fibroblastic appearance of MSCs with a similar cell number to that of the untreated cells. Cells treated with concentration above 10 *μ*M showed apparent changes in the fibroblastic morphology and reduced cell number. Changes at higher concentrations may have been due to cell death and/or cell detachment (see Figure 3(a)). Based on these observations, 10 *μ*M concentration of benzamil was thus selected for our experiments. The unstrained cells grew in random arrangements on silicone chambers, whilst the strained cells appeared elongated and aligned perpendicular to direction of stretch (see Figure 3(b)). There were no obvious morphological differences observed in ENaC-inhibited hMSCs or non-ENaC-inhibited hMSCs.

### 3.3. Changes in ECM Production during Stretching

**Figure 4:**
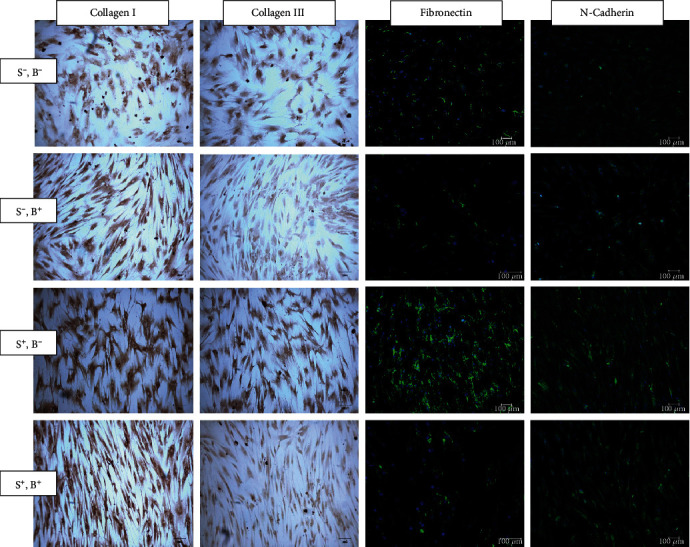
Immunostaining and immunofluorescence images of unstained and strained hMSCs cultured with or without benzamil. The cells were stained with immunostaining antibody collagen I and collagen III. Immunofluorescence was assessed on antibody fibronectin and N-cadherin. Each cell was stained with Hoechst (blue) to reveal the nucleus, and the images were merged with the corresponding fibronectin or N-cadherin (green). S^−^: no mechanical stimulation; S^+^: cyclic stretching applied; B^−^: no benzamil; B^+^: with ENaC inhibitor, benzamil.

Figure 4 shows the expression of collagen I, collagen III, fibronectin, and N-cadherin following immunostaining of the cells in both unstrained and strained cells treated with or without benzamil. Expression of collagen and especially collagen III was found to be slightly decreased in both unstrained and strained cells treated with benzamil as compared to cells without benzamil treatment. The expression of fibronectin and N-cadherin was increased in strained cells compared to unstrained cells; however, their expressions were reduced when treated with benzamil.

### 3.4. Influence of ENaC Inhibition on Tenogenic Differentiation

**Figure 5:**
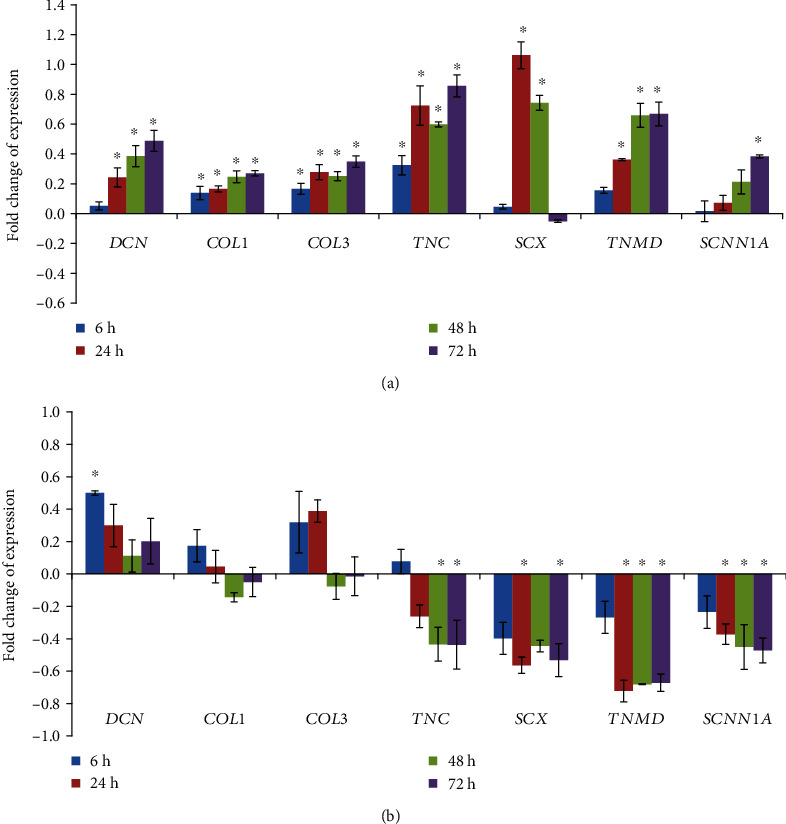
mRNA expression of tenogenic lineage genes and *α*-ENaC gene subjected to cyclic tensile loading. (a) Tenogenic differentiation of hMSCs is triggered by mechanical stimulation (1 Hz and 8% strain). The expression level of each gene was normalized with the level of housekeeping gene. Fold changes of expression were counted by normalizing to the relative expression amount of corresponding control groups (unstrained groups). Statistical significance (*p* < 0.05) was represented by ∗ compared to unstrained. (b) Tenogenic lineage genes' (*DCN*, *COL1*, *COL3*, *TNC*, *SCX*, and *TNMD*) expression was influenced after adding benzamil to the strained cells. The value of fold change was presented as the ratio of the strained group treated with benzamil to the strained group without benzamil. Statistical significance (*p* < 0.05) was represented by ∗ compared to the strained group without treatment. Error bars represent the SD of the mean of six biological replicates.

Our previous study shows that mechanical stimulation can trigger tenogenic differentiation of hMSCs [26]. From 6 hours to 72 hours, the expression of tenogenic markers appeared to be upregulated, with the exception of scleraxis, which was present at a higher level at 24 hours but decreased at later time points (see Figure 5(a)). The correlation between *α*-ENaC and hMSC tenogenic differentiation through mechanical stretching is analysed and presented in Table 2. Regression analysis showed that there was a strong positive correlation between *α*-ENaC expressions with tenogenic markers (with time), except for *SCX*. We then evaluated the effect of ENaC inhibition on mechanical strain-mediated tenogenic differentiation of hMSCs.

Blocking ENaC in cells subjected to mechanical loading resulted in a significant decrease in the expression of tenogenic markers (see Figure 5(b)). Although the expression of ECM components such as *DCN*, *COL1*, and *COL3* appeared to be increased during the earlier time points, these effects were diminished over time. The expressions of tenogenic markers were consistent with the immunostaining results of collagen (see Figure 4). *α*-ENaC gene (*SCNN1A*) was downregulated following ENaC inhibition. A drop in specific tenogenic gene expression including *TNC*, *SCX*, and *TNMD* was also observed. These observations support our hypothesis that ENaC (or more specifically *α*-ENaC) plays a vital role in the tenogenic differentiation processes following mechanical loading.

## 4. Discussion

ENaC, as an ion channel, has been shown to be a potent mechanotransducer in various cell types [27–29], and the mechanoactive role of ENaC particularly on the terminally differentiated cells appears to be well-established [7, 30, 31]. However, to the best of our knowledge, there have not been previous studies demonstrating the role of ENaC in regulating the mechanical strain-mediated tenogenic differentiation of hMSCs. This study is potentially the first to provide evidence of the involvement of ENaC on the mechanotransduction process that underpins the progression of hMSC differentiation in response to mechanical strain.

Previous studies have indicated the existence of four subunits (*α*, *β*, *γ*, and *δ*) of ENaC in human tissue or cells [32, 33]. Although all subunits were expressed in hMSCs, only *α*-ENaC appears to be related to the effects of stretching in hMSCs. From previous studies, *β*- and *γ*-ENaC have been shown to play an important role in mechanotransduction only in neurons innervating the aortic arch and vascular smooth muscle; and therefore, the lack of expression of these subunits in hMSCs is not unexpected [34]. Nevertheless, chondrocytes being cells of mesenchyme origin appears to response to mechanical signals through the propagation of signalling cascades initiated by the influx of sodium through mechanosensitive *α*-ENaC channels [11]. Hence, our finding of *α*-ENaC changes in hMSCs being responsive to stretching correlates well to the observations made in previous studies [35, 36].

We found that during mechanical stimulation, the expression of the functional subunit of *α*-ENaC increases in tandem with the increase in the expression of tenogenic differentiation markers. This apparent change in ENaC subunit stoichiometry during differentiation may suggest a specific role for the *α*-subunit of ENaC in the initiation and propagation of tenogenesis in hMSCs. Nevertheless, this does not indicate the lack of importance of the other subunits in this process. It merely suggests that *α*-ENaC is highly expressed during cell stretching; and based on previous studies, extracellular loops of other ENaC subunits may function as the sensors of mechanical loading that transmit the signal to the channel gating region, thereby enabling *α*-ENaC to function effectively [37, 38]. This complex interaction of the carboxyl terminal region of the *α*-ENaC to the actin cytoskeleton is thus necessary to propagate ENaC function, i.e., activating and proliferating the tenogenic differentiation process [38]. It is also worth noting that the subunits of ENaC may be enhanced by actin-disrupting agents or by addition of short actin filaments *in vitro* [37].

Another point worth mentioning is the fact that other studies suggest that certain ENaC subunits appear irrelevant for cellular function [39, 40]. Although it is reported that all subunits of ENaC contribute to the formation of functional channels [41], the existence of homomeric channels of *α*-ENaC alone with distinct properties was also found in some studies [33, 42]. In fact, similar studies appear to show a single upscaling of this subunit to produce a small amount of amiloride-sensitive currents suggesting the functionality of homomeric *α*-ENaC [43]. In a study expressing recombinant *α*-ENaC in stretch-activated cation channel, null cells of human primary osteoblast demonstrated increased nonselective cation channel activity, with an increase in channels permeable to calcium ions [44].

We can therefore conclude that although the other subunits may not have a direct role in tenogenic expressions observed, its presence is necessary for the tenogenic process to be initiated and propagated. Using the ENaC inhibitor benzamil, we were able to demonstrate this apparent observation, albeit benzamil is not a specific blocker of a specific ENaC subunit. Thus, the use of benzamil itself is insufficient to prove that *α*-ENaC is completely involved in the tenogenic process occurring during mechanical stretching. Furthermore, sodium channel blockers had demonstrated the inhibitory effect on collagen accumulation in extracellular matrix [45]. This may explain the observed decrease in collagen in our experiments on treatment with benzamil. With ENaC blocking and reduced Na^+^ influx, the expression of tenogenic markers was also reduced dramatically.

**Figure 6:**
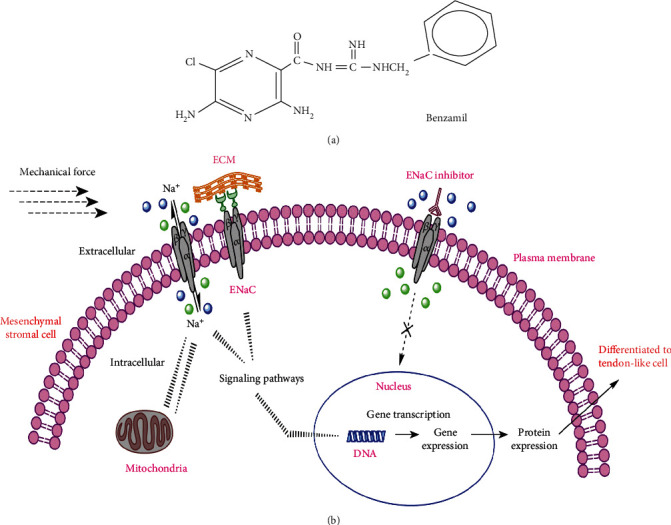
Proposed mechanism for the regulation of hMSCs tenogenic differentiation signaling pathways by ion channel ENaC. (b) ENaC leads to upregulation of the tenogenic gene markers, which in turn activates the tenogenic differentiation signaling pathway. Benzamil (a) which is the ENaC inhibitor, inhibits this pathway, and thus influences the cell differentiation.

In the present study, the use of benzamil, a specific inhibitor to ENaC but not of its subunits, was chosen instead of amiloride. This was so since it has been suggested, albeit with some sense of lack of conviction, that benzamil is more effective in limiting the adverse effect of ENaC blockage on cell viability [46]. The blocking effect of benzamil appears to result from the benzene ring at the guanidino end of the molecule (see Figure 6(a)) [47], which is deemed to be molecule specific. Furthermore, amiloride has been shown to interfere with several cellular pathway processes, including inhibiting the Na^+^/H^+^ exchanger mechanisms [48]. Benzamil on the other hand is more stable and has a very high affinity for the Na^+^ channel without affecting other major channels including K^+^ channels [49, 50]. It has been suggested that the ionic channel block using benzamil at 1 *μ*mol L^−1^ results in a complete halt of cellular function and can only be partially reversed. Hence, in minimal amounts, the effect of ENaC blocking can be better appreciated without the need to change the volume of the culture media.

There are several studies using inhibition of certain gene expression with siRNA (small interfering RNA) approach. Whilst the use siRNAs is an option for gene knockdown experiment, it has several issues which need to be taken into consideration such as nonspecific and incomplete silencing. In addition, it has been reported that the transfection ability of the primary cells is limited as compared to cancer cells, and the RNases will be actively engaged in degrading and eliminating the transfected siRNA. This will result in transient inhibition of the siRNA effect as the molecule is active only for a short time [51]. Besides, the transcripts with high turnover are sometimes difficult to silence. Thus, the use of this technique may not be the best choice in this present study.

It has been demonstrated that there are three possibilities as to how ENaC channels can be activated or blocked [52, 53]: (1) by controlling the bilayer tension or curvature directly activating the channel; (2) by controlling the release of another molecule from a cell that in turn activates the channel, for example, in the case of the present study where benzamil works by preventing sodium from moving intracellularly and competitively inhibiting sodium influx; and (3) by activating a tethering mechanism in which the ion channel binds either to the cytoskeleton or to the extracellular matrix. It has also been suggested that ENaC may perform other functions in MSCs, just like those of degenerins [54]. In our study, it was mostly the unstrained hMSCs that express low levels of ENaC. A rise in intracellular sodium activates C-Jun NH2-terminal kinase/stress-activated protein kinase (JNK), a member of the mitogen-activated protein kinase (MAPK) family, and the stress-activated protein kinase (SEK1) [55]. SEK1 can phosphorylate and activate JNK, which in turn phosphorylates C-Jun leading to an increased transcriptional activity. Thus, alterations in intracellular sodium concentration could trigger a cascade of transduction signals ultimately interfering with tenocyte-specific transcription factors. In contrast, if the ENaC of the cells is inhibited, there is no balance between extracellular and intracellular sodium concentration thus activating the signalling pathways and influencing tenogenesis expression. This process is illustrated in Figure 6.

Although the current study is robust in its design and provides us with a valuable insight into the role of *α*-ENaC in hMSC differentiation, there were limitations which were unavoidable but are worth highlighting. To directly investigate elevated ENaC activity, the strained and unstrained hMSCs (either block by benzamil or not) should be subjected to whole-cell patch clamp recordings to analyse the benzamil-sensitive currents. We hope that by implementing this technique in the future, we will able to demonstrate that the ENaC/degenerin family of proteins is capable of mediating both transepithelial sodium transport and is directly responsible for the process of mechanotransduction. Secondly, the present study also did not study the involvement of other ionic fluxes, which, as many would concur, is a dynamic process of interrelated ionic interaction that may contribute to the tenogenic differentiation process.

The findings in this study nevertheless elucidate the roles of biomechanical stimulation and ion channel on hMSC differentiation towards a tendon fibroblast phenotype. As stated earlier, experimental control over progenitor cell lineage specification can be achieved by modulating properties of the cellular microenvironment. Understanding the microenvironments in which the MSCs reside and differentiate *in vivo* and trying to recapitulate these *in vitro* to further control stem cell differentiation has become an increasingly important area of stem cell research. Besides mechanical stimulation, other strategies including the use of soluble factors, ECM proteins, and biomaterials may also play an important role in hMSC differentiation. Several studies have shown that scaffolds (e.g., bioactive nanofibers and rope-like silk scaffolds) and growth factors (e.g., GDF5 and GDF7) can activate multiple signalling cascades, including MAPK, ERK, and Rho/ROCK, ultimately leading to the MSC tenogenic differentiation [56, 57]. Therefore, the application of growth factors and scaffolds in combination with mechanical stimuli may synergistically enhance tenogenic differentiation of hMSCs through amplification of the signalling pathways. However, the interactions between biochemical and mechanical cues in directing hMSC differentiation towards tenogenic lineage are still not fully understood and remain to be explored. Therefore, the focus of future studies could be directed in investigating the mechanisms underlying the synergistic effect of the biochemical and mechanical signals in influencing cell fate. With a better understanding of this process, incorporation of growth factors and/or scaffold in combination with mechanotransduction may constitute a novel approach to achieve successful tendon tissue engineering via effective regulation of cellular differentiation.

## 5. Conclusions

The present study demonstrated that (1) although *α*, *β*, *γ*, and *δ* subunits of ENaC were expressed in hMSCs, only the expression of the functional *α* subunit is higher during stretching at 1 Hz and 8% strain, thus suggesting that *α*-ENaC is the main mechanosensitive ion channel that influences tenogenic differentiation of hMSCs, (2) uniaxial strains at 8% is required to elicit significant tenogenic expressions, and (3) there is a positive correlation between the *α*-ENaC expression and tenogenic marker expressions which is altered in the presence of ENaC blocker benzamil, thus strengthening our hypothesis that ENaC (and more specifically the *α*-ENaC subunit) may be implicated in regulating the tenogenic differentiation process of hMSCs during cell stretching.

## Figures and Tables

**Table 1:** The genes of interest determined in this study.

Related marker	Gene name	Abbreviation
ENaC subunit	Sodium channel, nonvoltage-gated 1, alpha	*SCNN1A*
	Sodium channel, nonvoltage-gated 1, beta (Liddle syndrome)	*SCNN1B*
	Sodium channel, nonvoltage-gated 1, delta	*SCNN1D*
	Sodium channel, nonvoltage-gated 1, gamma	*SCNN1G*

ECM component	Collagen type I, *α*1	*COL1*
	Collagen type III, *α*1	*COL3*
	Decorin	*DCN*

Tendon lineage	Tenascin C	*TNC*
	Scleraxis homolog A	*SCX*
	Tenomodulin	*TNMD*

Housekeeping gene	Phosphoglycerate kinase 1	*PGK1*

**Table 2:** Regression analysis of the relationship of *α*-ENaC with different cell lineage genes after mechanical strain.

Gene	Positive or negative correlation (*R*^2^)
*COL1*	+0.8762
*COL3*	+0.8761
*DCN*	+0.9557
*TNC*	+0.7843
*SCX*	-0.0253
*TNMD*	+0.8318

## Data Availability

The data used to support the findings of this study are included within the article.
